# Characteristics of gene expression in epicardial adipose tissue and subcutaneous adipose tissue in patients at risk for heart failure undergoing coronary artery bypass grafting

**DOI:** 10.1186/s12864-024-10851-9

**Published:** 2024-10-07

**Authors:** Christoffer Frisk, Mattias Ekström, Maria J Eriksson, Matthias Corbascio, Camilla Hage, Hans Persson, Cecilia Linde, Bengt Persson

**Affiliations:** 1 Department of Cell and Molecular Biology, Science for Life Laboratory, Uppsala University, Box 596, Uppsala, S-751 24 Sweden; 2 Department of Clinical Sciences, Karolinska Institutet, Danderyd Hospital, Stockholm, S-182 88 Sweden; 3 Department of Cardiology, Danderyd Hospital, Stockholm, S-182 88 Sweden; 4 Department of Clinical Physiology, Karolinska University Hospital, Stockholm, S-171 76 Sweden; 5 Department of Molecular Medicine and Surgery, Karolinska Institutet, Stockholm, S-171 77 Sweden; 6 Department of Thoracic Surgery, Karolinska University Hospital, Stockholm, S-171 76 Sweden; 7 Department of Medicine, Karolinska Institutet, Stockholm, S-171 77 Sweden; 8 Karolinska University Hospital, Heart and Vascular Theme, Stockholm, S-171 76 Sweden

**Keywords:** Epicardial adipose tissue, Gene expression, Weighted gene cluster, Heart failure, Bioinformatics

## Abstract

**Background:**

Epicardial adipose tissue (EAT) surrounds the heart and is hypothesised to play a role in the development of heart failure (HF). In this study, we first investigated the differences in gene expression between epicardial adipose tissue (EAT) and subcutaneous adipose tissue (SAT) in patients undergoing elective coronary artery bypass graft (CABG) surgery (*n* = 21; 95% male). Secondly, we examined the association between EAT and SAT in patients at risk for HF stage A (*n* = 12) and in pre-HF patients, who show signs but not symptoms of HF, stage B (*n* = 9).

**Results:**

The study confirmed a distinct separation between EAT and SAT. In EAT 17 clusters of genes were present, of which several novel gene modules are associated with characteristics of HF. Notably, seven gene modules showed significant correlation to measures of HF, such as end diastolic left ventricular posterior wall thickness, e’_mean_, deceleration time and BMI. One module was particularly distinct in EAT when compared to SAT, featuring key genes such as FLT4, SEMA3A, and PTX3, which are implicated in angiogenesis, inflammation regulation, and tissue repair, suggesting a unique role in EAT linked to left ventricular dysfunction. Genetic expression was compared in EAT across all pre-HF and normal phenotypes, revealing small genetic changes in the form of 18 differentially expressed genes in ACC/AHA Stage A vs. Stage B.

**Conclusions:**

The roles of subcutaneous and epicardial fat are clearly different. We highlight the gene expression difference in search of potential modifiers of HF progress. The true implications of our findings should be corroborated in other studies since HF ACC/AHA stage B patients are common and carry a considerable risk for progression to symptomatic HF.

**Supplementary Information:**

The online version contains supplementary material available at 10.1186/s12864-024-10851-9.

## Introduction

The relationship between epicardial adipose tissue (EAT) and cardiac function has generated significant interest in past years. Recent advances have highlighted the role of EAT in systemic inflammation and its association with metabolic syndromes, underscoring its importance beyond fat storage [1]. This study aims to build upon these discoveries by exploring whether and how gene expression in EAT might influence cardiac physiology.

EAT is located between the myocardium and the pericardium, and is primarily composed of adipocytes that are generally smaller than those in other adipose tissues and has unique characteristics. EAT's anatomical proximity to the heart allows it to exert both beneficial and detrimental effects directly on the cardiac muscle, as well as the coronary arteries through paracrine and vasocrine mechanisms. Importantly, the epicardium shares a microcirculation with the underlying myocardium, and under healthy conditions EAT produces cytokines that support the heart [2]. However, in the presence of chronic inflammatory disorders [3], EAT, being a rich source of free fatty acids, cytokines, and adipokines [4, 5], may induce inflammation, contribute to atherosclerosis [6], atrial and ventricular fibrosis and play divergent roles in the pathophysiology and development of HF [7–9]. Approximately 60–70% of the ATP necessary for cardiac muscle contraction [10] is generated through mitochondrial oxidation of fatty acids. Under normal physiological conditions, EAT serves multiple functions: it acts as a buffer by absorbing fatty acids, thereby shielding the myocardium from excessive fatty acid levels, and it serves as an energy reserve in the form of lipid storage [4]. Systemic inflammation has been shown to activate the nuclear factor-κB regulatory pathway in EAT in addition to omental and subcutaneous adipose tissue [11], potentially exacerbating cardiovascular conditions. Moreover, studies have indicated that an increase in EAT size is linked to the onset and progression of coronary artery disease [12], and has been identified as a potential predictor of the disease [13]. This link between EAT and cardiovascular health highlights an important area for further research. The link underscores the importance in examining the molecular mechanisms through transcriptomic data where much is still to be discovered in regards to which genes and pathways that are driving these conditions.

In our previous research as part of the CABG-PREFERS [14] study, we have examined samples from the left and right ventricles (LV and RV) in relation to tissue and HF phenotype [15, 16]. Building upon this, our current work was extended to study EAT. Our definition of HF in this work is based on ACC/AHA/HFSA criteria on stages of HF, which includes stage A, at risk of developing heart failure, and stage B, implying no current or previous symptoms of heart failure but with structural echocardiographic changes, signs of elevated filling pressures and/or elevated NT-proBNP [17]. The aim of the present study was to compare gene expression between epicardial adipose tissue (EAT) and subcutaneous adipose tissue (SAT) using RNA sequencing, and linking the genetic expression with echocardiographic data. Additionally, we have investigated differential expression between various HF phenotypes within EAT itself. This extension allows us to better understand the unique roles of EAT in the context of cardiac dysfunction, and how these roles may vary across different clinical presentations of HF.

## Results

### Patients

Our study is based on 21 patients from whom EAT and SAT samples were acquired during elective coronary artery bypass surgery [14]. The cohort predominantly comprised males (20/21, 95%), with a median age of 65 years (Q1: 61.5 to Q3: 72.5) and a median Body Mass Index (BMI) of 27.1 (Q1: 24.9, Q3: 29.7) (Table 1).

**Table 1:** Patient characteristics. Data are expressed as median and quartiles (Q1;Q3) or number (%)

	**Stage (*n*** **= 12)**				**Stage B (***n* **= 9)**				
**Variable**	**Median**	**Q1**	**Q3**	**Sample**	**Median**	**Q1**	**Q3**	**Sample**	***P*-Value**
Age, years	63	59.5	72.5	12	70	64	72	9	0.544
Weight, kg	81	75.0	92.3	12	87	84	110	9	0.081
Height, cm	179	169.5	182.3	12	179	176	185	9	0.498
Body mass index, kg/m2	27	24.4	29.4	12	26	25	33	9	0.414
	***n***	**%**			***n***	**%**			***P*-value**
Sex (male)	10	83			9	100			0.59
Smoking, previous	5	42			3	33			1.00
Smoking, present	0	0			0	0			
Atrial fibrillation	2	17			5	56			0.16
Hypertension	8	66			9	100			0.17
Diabetes	4	24			6	67			0.28
Peripheral vessel disease	1	8			0	0			1.00
Stroke/TIA	1	8			1	11			1.00
Previous percutaneous coronary intervention	2	17			1	11			1.00
**HF status and Physical findings**	***n***	**%**			***n***	**%**			
NYHA class I	7	58			3	33			
NYHA class II	5	42			6	67			
	**Median**	**Q1**	**Q3**	**Sample**	**Median**	**Q1**	**Q3**	**Sample**	***P*-Value**
Systolic blood pressure, mmHg	129	123	142	12	139	128	158	9	0.337
Diastolic blood pressure, mmHg	75	68	79	12	74	64	82	9	1.000
Heart rate, beats/min	64	60	67	12	70	62	80	9	0.144
eGFR ml/min/1.73m2	89	72	91	11	80	58	88	8	0.492
Creatinine, Âµmol/l	79	78	90	11	90	81	116	8	0.200
NT-proBNP, ng/l	182	86	253	10	578	285	731	8	0.003
Potassium, mmol/l	4.1	4	4	12	4	4	5	9	0.171
Sodium, mmol/l	141	140	142	12	140	139	142	8	0.435
Uric acid, Âµmol/l	320.5	294	385	10	406	357	440	7	0.097
LDL, mmol/l	1.55	1	2	10	2	2	2	8	0.285
hsTroponin T, ng/l	16	9	19	10	15	10	23	8	0.964
HbA1c, mmol/mol	40	36	46	10	50	39	59	9	0.253
Glucose, mmol/l	6.6	5	8	11	9	6	11	7	0.123
**Treatment**	***n***	**%**			***n***	**%**			***P*-value**
Antiplatelet	11	91			6	67			0.377
Anticoagulant	0	0			1	11			0.882
Nitrates	2	17			2	22			1.000
Beta blocker	8	67			8	89			0.505
ACE inhibitor	2	17			3	33			0.712
ARB	3	25			3	33			1
Statin	10	83			8	89			1

*ACE* Angiotensin converting enzyme, *ARB* Angiotensin II receptor blocker, *BMI* Body Mass Index, *NT-proBNP* N-Terminal pro-Brain Natriuretic Peptide

### Sequencing

The mapping of sequencing reads was of high quality, with an average of approximately 34.95 million uniquely mapped reads per sample (standard deviation: 4.21 million) (Supplementary Figure S1a). The sample concentration was too low to calculate RIN values. However, the gene body coverage analysis demonstrated consistent coverage across most of the transcript, indicating that RNA degradation did not introduce significant biases (Supplementary Figure S1b).

### Difference in gene expression between EAT and SAT

**Fig. 1:**
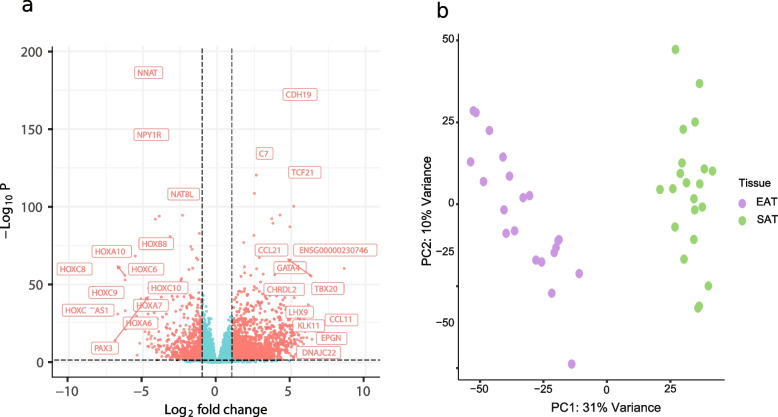
**a** Volcano plot displaying differentially expressed genes between EAT and SAT with x axis representing log_2_ fold change and y axis the adjusted *p*-value. Significant (FDR adjusted *p*-values ≤ 0.05) genes with |log_2_FC|> 0.5 coloured in orange. **b** PCA score plot showing sample distribution over component 1 and 2 in x- and y-axis, respectively. Epicardial samples are coloured blue and subcutaneous orange

The study included 42 paired biopsies (EAT *n* = 21 + SAT *n* = 21) from 21 patients. To assess differences in gene expression and function between within EAT and between EAT and SAT, a combination of differential expression analysis and network clustering was applied to normalised counts data. The extent of differentiation between the two tissue types was represented by using absolute value of the log_2_ fold change (FC). Transcriptome profiles for each patient and tissue group were generated using DESeq2, while the cluster analysis was performed employing WGCNA. We then conducted functional cluster analysis using Enrichr, focusing specifically on Gene Ontology (GO) and KEGG pathway analyses. Additionally, we analysed correlations with echocardiographic parameters and identified key cluster hub genes. The analysis of gene expression profiles revealed significant differences in the first principal component, with 8626 differentially expressed genes (DEGs) at a false discovery rate ≤ 0.05. Filtering on genes exhibiting a log_2_ fold change of less than –1 or greater than + 1 resulted in identifying 1,191 upregulated and 508 downregulated genes. The volcano plot in Fig. 1a shows the most significant up- and down-regulated genes in EAT versus SAT. The genes that were most up-regulated, listed in descending order of log_2_FoldChange, were TBX20, ALOX15, CCL21, CHRDL2, KLK11, ANXAB, IGLV3-25, GATA4, TCF21 and CDH19. Among the ten most down-regulated genes in EAT versus SAT, seven were HOX genes, four of which were from the HOXC family: HOXC8, HOXC-AS1, HOXC9, and HOXC6. The remaining genes, listed in descending order of foldchange value, were HOXA10, HOXB8, HOXA6, ENSG00000260597, EN1 and NNAT.

We conducted a principal component analysis (PCA), an unsupervised classification method, to assess the relationship between EAT and SAT. The PCA showed that there was a clear separation between EAT and SAT samples along the first component. This shows that they have distinct characteristics that can be detected based on their gene expression patterns (Fig. 1b).

### Adipokines in epicardial adipose tissue

Notable pro-inflammatory genes with significant up-regulation in EAT vs. SAT were IL18, CXCL1, CCL11, TLR2, and CCL5, while those with down-regulation were PTGS2, IL6 and CXCL8. Typical adipocyte genes LEP, ADIPOQ, IL6, SERPINE1 and RBP4 showed a significant down-regulation in EAT while ITLN1 expressed an up-regulation. Intersection of significant differentially expressed adipose specific genes [18] revealed an overlap of 38 genes. Most genes showed a skewed expression profile towards lower levels in EAT, with the exception of IL2RA, PRG4, ITLN1 and UPK3B which had higher expression in EAT.

### Gene expression difference in EAT comparing ACC/*AHA* stage A vs. stage B

For the purpose of this analysis, differential gene expression analysis comparing EAT of ACC/AHA stage A (*n* = 12) with stage B (*n* = 9) was conducted. The analysis revealed 18 significant genes (*p*-value ≤ 0.05); 11 of these were up-regulated and 7 were down-regulated in stage B relative to stage A. A full list of the up-regulated and down-regulated genes can be found in Supplementary Table 1, which include the up-regulated SPP1, CHIT1, MMP7, TUBB2B, and ADAM12 and the down-regulated ITGA9, GRIA2, IGKV2-40, and PDLIM3.

### Weighted gene correlation network modules in EAT

The weighted gene correlation network was calculated on the entire EAT dataset with SAT as reference. A soft-threshold power was introduced to the network correlations affecting the topology and where an approximate scale-free topology was found for β = 18 (R^2^ > 0.8) A hierarchical clustering was conducted based on these weighted correlations. In the dendrogram of gene-to-module associations, genes that were strongly correlated in terms of their expression levels were grouped together into modules. To simplify downstream analysis, modules that were similar to each other were merged. This reduced the total number of 35 modules to a final count of 17. With this merging, the original numbering system of the modules has been restructured. Initially, the modules were numbered based on the numeral of their gene members, with the largest module assigned the first number. After the merging process, the module numbers do not follow the original order.

**Fig. 2:**
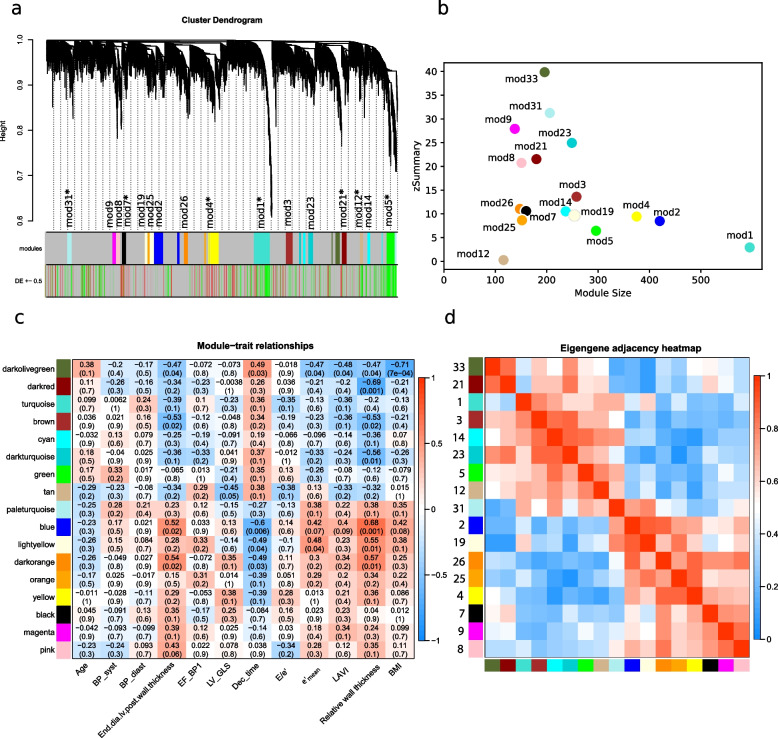
**a** Gene cluster dendrogram with height representing 1–biweight midcorrelation in the y axis. Gene module membership is represented by colour in the first row with unassigned genes in grey. The second row show the differential expression of genes between EAT and SAT, where genes with a log2FC above 0.5 are shown in green, and those below –0.5 in red. Modules with significant (*p* ≤ 0.05) enrichment displayed with asterisks. **b** Scatter plot visualisation of module preservation represented in with module size in x axis and zSummary score in y axis. **c** Pearson correlation between modules and echo parameters. Heatmap displays correlation values with p-values within parentheses. Correlations are coloured red and blue for negative and positive correlations, respectively. Echo variables with no significant correlation in any module were omitted. **d** Eigengene correlation matrix over all modules ranging from no correlation (0) to strong correlation (1)

The gene to module dendrogram of the EAT samples with highlighted EAT vs SAT DEGs is shown in Fig. 2a. The EAT module preservation, a measure of how well a module's structure is maintained in different data sets, was calculated based on network density and connectivity with reference to SAT. The zSummary score ranges from 0.29 (module 12) to 43 (module 33) as shown in Fig. 2b. Module correlation to echocardiography parameters showed significant numbers for end diastolic left ventricular posterior wall thickness, deceleration time, e’_mean_, and BMI, over 7 modules (Fig. 2c). Each module’s eigen gene, its intramodular principal component, was calculated and used to correlate both genes within the module as well as other modules. For the latter the eigen gene adjacency matrix module revealed two larger clusters consisting of 9 and 8 modules, respectively (Fig. 2d).

Cross-referencing the gene–gene connections in the modules to the STRING database of protein–protein interactions shows overlap among the top five for module 2 (blue) (82%), module 1 (turquoise) (78%), module 31 (pale turquoise) (74%), module 4 (yellow) (74%) and module 19 (light yellow) (72%). With the exception of module 1, these categories have an average combined score above 0.6, proving medium to strong connections. Module 8 (pink) showed least overlap with only 4%. The STRING networks can be found in Supplementary Figure S2.

**Fig. 3:**
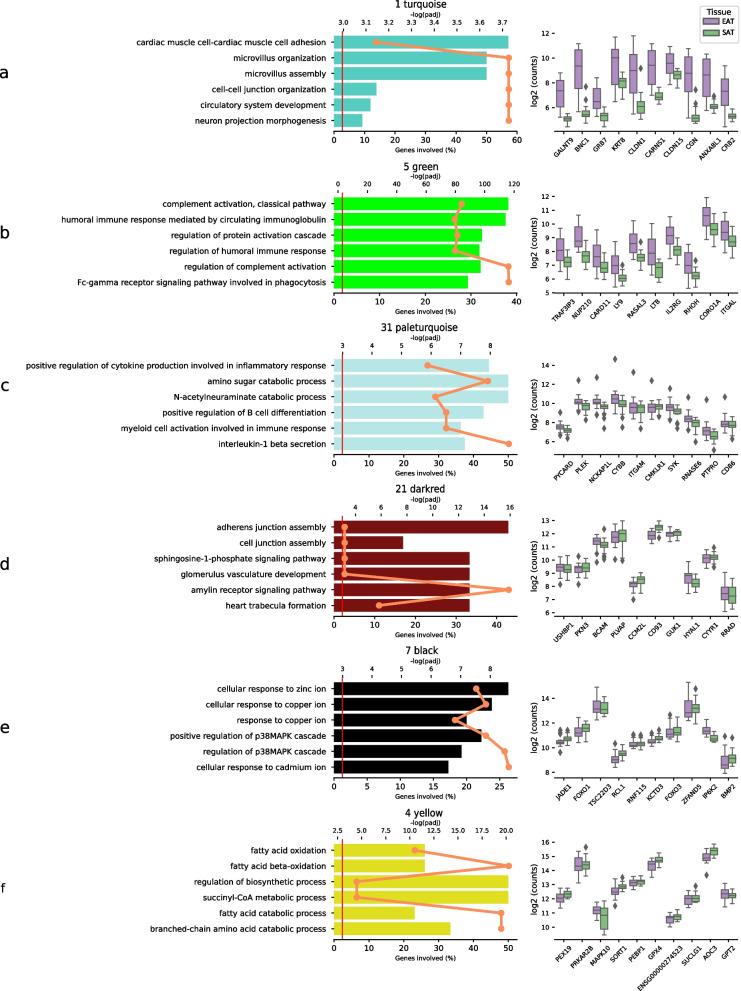
Enrichment analysis and top hub gene expression levels. All modules in the figure contains enrichment for DE genes. The bar plot displays GO enrichment for each term, with the top x-axis representing the –log(padj) values plotted as a line and the bottom x-axis indicating the percentage of involved genes. The GO terms are ordered by EnrichR's combined score. Adjacent to the GO plot, the box plot presents the expression levels in the top 10 highest-ranked hub genes within the module

**Fig. 4:**
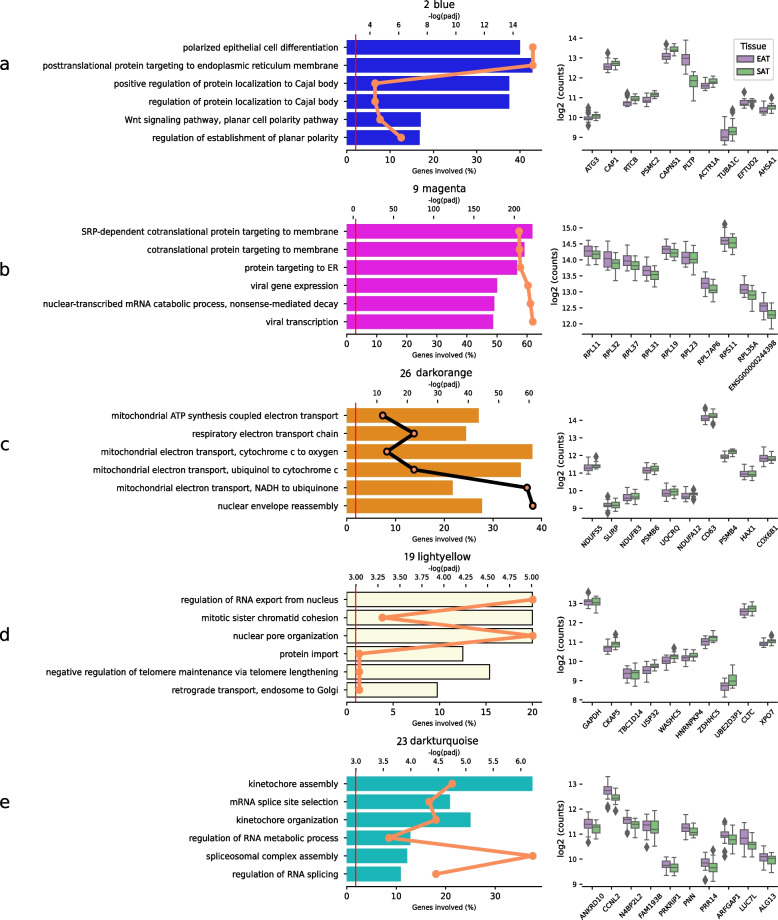
Enrichment analysis and top hub gene expression levels of modules with no significant enrichment of DE genes. The bar plot displays GO enrichment for each term, with the top x-axis representing the –log(padj) values plotted as a line and the bottom x-axis indicating the percentage of involved genes and the top x-axis. The GO terms are ordered by EnrichR's combined score. Adjacent to the GO plot, the box plot presents the expression levels in the top 10 highest-ranked hub genes within the module

The weighted correlation analysis of the EAT data revealed 17 gene modules of which five were enriched with upregulated DEGs; module 1 (turquoise), module 5 (green), module 12 (tan), module 21 (dark red) and module 31 (pale turquoise) and three modules enriched with downregulated DEGs; module 7 (black), module 4 (yellow) and module 21 (dark red). These enrichments are indicated by asterisks in Fig. 2a. Module preservation was overall high; 10/17 modules exhibited a zSummary score above 10 and only one falling below 2. The most preserved modules were; module 33 (dark olive green), module 31 (pale turquoise), module 23 (dark turquoise), module 9 (magenta) and module 21 (dark red) while module 12 (tan) was the least preserved module (Fig. 2b). In addition to module preservation, GO enrichment within these modules was also examined to get insight into the biological processes these genes may be involved in. Eleven modules showed significant GO enrichment after adjustment for multiple testing, suggesting these groups of genes are involved in distinct biological processes or pathways (Figs. 3 and 4). The specific enrichments and hub genes for each module are detailed in the following sections, presented in the order they appear in the accompanying figures. Additionally, gene-to-module membership details are available in Supplementary Table S2 as well as extended GO enrichment in Supplementary Figure S3.

Module 1 (turquoise) was the largest with 595 members containing 372 significantly upregulated genes in EAT vs SAT, whereof the most upregulated were: ALOX15, KLK11, ANXA8, PKHD1L1, CGN and ITLN1 (Supplementary Table S2). Enriched genes were involved in cardiac muscle cell adhesion, such as DSP, CXADR and DSG2, as well as microvillus assembly, including SLC9A3R1, STK26, TNIK and EZR. Of the most connected hub genes, nine of ten, except for GALNT9, were significantly upregulated in EAT, among which CGN and BNC1 obtained the highest fold change (Fig. 3a).

Module 5 (green) had a strong enrichment of genes related to immune-related processes; complement activation, classical pathway, humoral immune response mediated by circulating immunoglobulin, regulation of the protein activation cascade, regulation of the humoral immune response, and regulation of complement activation (Fig. 3b). All of the most highly connected hub genes were significantly upregulated, including NF-κB activating CARD11, the transport channel protein NUP210 controlling muscle differentiation12, and the Ras homolog family member RHOH. KEGG pathway enrichment analysis identified the T Cell Receptor Signalling and Th1 and Th2 cell differentiation pathways, Supplementary Figure S4.

Module 31 (pale turquoise) was associated with cytokine production involved in inflammatory response (GO:0002534) through GBP5, TLR6, TLR4, and MYD88 (Fig. 3c). Furthermore, the module showed enrichment for amino sugar catabolic process (GO:0046348) through GNPDA1, NAGK, and RENBP.

Module 21 (dark red) exhibited an enrichment of genes involved in maintaining cellular structure, including those involved in the assembly of adherens and cell junctions (Fig. 3d). This module also displayed an enrichment in the amylin receptor signalling pathway (GO:0097647), a pathway integral to metabolic regulation, including the balance of glucose in the blood [19].

Module 7 (black) was enriched with genes involved in the cellular response to zinc ions (GO:0071294) and the regulation of the p38MAPK cascade (Fig. 3e). The genes involved in the latter are PER1, GADD45B, GADD45G, HAND2 and the hub gene BMP2.

Module 4 (yellow) with 375 members was enriched with genes of fatty acid oxidation (GO:0019395) and cellular response to peptide hormone stimulus (GO:0071375) and downregulation of ADIPOQ, PPARG, CAV1, ENPP1 and hub gene PEX19 (Fig. 3f). With an overlap of 26/196 module 4 contained the largest enrichment of adipose specific genes, according to the Human Protein Atlas [18] (Supplementary Table S2).

Module 2 (blue) contained genes enriched in the processes of polarised epithelial cell differentiation (GO:0030859) and neutrophil degranulation (GO:0043312), including the hub genes PSMC2 and CAP1 (Fig. 4a). The module also contained the hub genes PLTP and CAPNS1, significantly upregulated in EAT. The module shows strong correlation to relative wall thickness (0.68, *p*-value:0.001) and negative correlation to deceleration time (–0.6, *p*-value:0.006) (Fig. 2c).

Module 9 (magenta) was primarily composed of ribosomal protein coding genes (84/138) and gene enrichment of SRP-dependent co-translational protein targeting (55/89), including hub genes such as RPL23, RPS25, RPL32, RPL31, RPL37, RPS6, and RPL11 (Fig. 4b).

Module 26 (dark orange), module 19 (light yellow) and module 23 (dark turquoise) were characterised by an enrichment of genes associated with fundamental cellular functions (Fig. 4 panels c, d, e). These functions involve processes such as mitochondrial ATP synthesis coupled electron transport, and regulation of RNA export from the nucleus and kinetochore assembly. Module 26, in addition to its association with the processes mentioned, also exhibits a link with cardiac structural characteristics, particularly showing a correlation with relative wall thickness and a negative correlation with deceleration time. Hub genes within the module include NDUFS5, NDUFB3 and NDUFA12 which encode for subunits of the mitochondrial complex I. The KEGG analysis of module 26 further revealed genes involved in Oxidative Phosphorylation and Diabetic Cardiomyopathy (Supplementary Figure S4).

Module 19 (light yellow) was involved in the nucleus functionality. Its primary activities include managing the transport of RNA from the nucleus to the cytoplasm. Additionally, it is involved in the organisation of nuclear pores, which are essential for the bidirectional transport of macromolecules (Fig. 4d).

Module 23 (dark turqoiuse) displayed an enrichment on biological processes critical for proper function of the cell cycle and gene expression with enrichment in kinetochore assembly and mRNA splice site selection.

Module 12 (tan), comprising 116 genes, was identified as the least preserved module (Fig. 2b). This module included the genes CCL21, TBX1, FLT4, ITLN1, RELN, ADAM23 and PTX3.

Module 33 (dark olive green) demonstrated correlations with BMI, end diastolic LV posterior wall thickness, LAVI, and e’_mean_, however not enriched for any significant GO terms. Among the hub genes in this module were SELENOH, NDUFB7, SCAND1, RAB40C and RAB11B. Notably, RAB11B and RAB40C, both part of the RAS oncogene family, play roles in vesicular trafficking. RAB11B, in particular, is linked to insulin secretion following a glucose stimulus (GO:0035773), while NDUFB7 is involved in the mitochondrial electron transport chain.

## Discussion

In our study, which included 21 individuals undergoing elective CABG due to stable coronary artery disease, with a predominantly male cohort (95%), we firstly uncovered significant variations in gene expression patterns between epicardial adipose tissue (EAT) and subcutaneous adipose tissue (SAT). Additionally, our research revealed a link between EAT and signs of heart failure, as evidenced by our echocardiographic indices, including LV wall thickness, e’_mean_, deceleration time and BMI. Moreover, we also observed distinct differences in gene expression between patients at risk for HF with no symptoms (ACC/AHA stage A) compared to those with signs of HF with no symptoms (ACC/AHA stage B).

### Differences in gene expression in EAT between ACC/*AHA* stage A and stage B

In our analysis, we observed significant differential gene expression in EAT when comparing stage A with stage B. Among the upregulated genes in stage B we found SPP1, CHIT1, MMP7, TUBB2B, and ADAM12. Increased expression levels of SPP1 are associated with several pathological heart states [20] and are elevated in the heart following myocardial infarction. It is believed to contribute to the subsequent remodelling process by promoting fibrosis [21] and affecting the overall tissue repair mechanisms. CHIT1 and MMP7 are involved in inflammation and matrix remodelling. The CHIT1 protein has been proposed as an early biomarker of atherosclerosis and is implicated in the innate immune system activation seen in HF, although further research is needed [22]. It has also been found to be a sex specific biomarker for new-onset HF in men, which can be an effect of the higher prevalence of atherosclerosis in male patients [23]. This observation may be the result of the predominantly male composition of our dataset. MMP7 enhances the breakdown of extracellular matrix components, potentially contributing to myocardial remodelling observed in both mild and severe HF states in rat models [24]. Tubulin isoforms, including TUBB2B, are up-regulated in early cardiac hypertrophy, which has suggested their involvement in reorganisation and stabilisation of the microtubule network essential for supporting the changes in cardiac cell architecture and function during hypertrophic changes [25]. Further, ADAM12 has been found to play a role in both stimulating and inhibiting cardiac hypertrophy, depending on the context and specific interactions with other proteins [26]. The increased expression could be a response to the mechanical and biochemical stresses experienced in HF conditions, where ADAM12 acts on various substrates including growth factors and integrins, influencing cell communication and extracellular matrix remodelling. Among the down-regulated genes in stage B, ITGA9 and PDLIM3 are found. ITGA9 codes for an integrin subunit. Integrins are transmembrane receptors important in several cellular processes such as migration, proliferation, survival, and differentiation, all important in cardiac remodelling and repair. However, a direct link to any cardiovascular outcome has yet to be established. Finally, PDLIM3 is member of a family of proteins that usually take part in organising the actin cytoskeleton. The protein is involved in linking actin filaments to Z-discs, which are crucial for transmitting mechanical forces across the cell and maintaining the structural integrity of cardiac muscle during both normal and stress-induced contractile function [27]. Notably, polymorphisms in PDLIM3 have been identified as contributing factors to the development of idiopathic dilated cardiomyopathy [28]. Differences in genetic expression of circRNA in EAT comparing HF vs non-HF individuals have been observed in another study (*n* = 10) where the changes in genes showed involvement in cell proliferation and inflammatory response [29].

While we can identify genes that potentially influence the myocardium in EAT in relation to the HF stage classification, it is notable that the genes likely to affect the myocardium – ADIPOQ, TNF-α, IL6, and PPARG – are not found in the set of DEGs. There are several potential explanations for this: either changes in these genes are not present between the phenotypes, it could be that these changes only arise in the later stages of the disease, the disease mechanisms driving HF may involve other pathways or genes that are more critical in EAT's role in HF, thus not highlighting these genes as differentially expressed, or the sample size may be too small to detect subtle changes in these gene expressions.

### Differences in gene expression between EAT and SAT across all phenotypes

In EAT, all phenotypes combined, the genes TBX20, GATA4, CCL21, CHRDL2, ALOX15, TCF21 (epicardin), and GRB7, are notably upregulated when compared to SAT. Among these genes, TBX20 and GATA4 are both involved in atrial septum morphogenesis and cardiac right ventricle morphogenesis [30, 31]. The cytokine CCL21 is involved in immune regulatory and inflammatory processes, inhibiting haematopoiesis and stimulating chemotaxis. CHRDL2 is typically expressed in smooth muscle and previously found enriched in myocardial tissue with hypertrophic cardiomyopathy [32]. ALOX15 has been shown to be highly expressed in ischemic heart tissue [33], it catalyses the conversion of arachidonic acid to 15-hydroxy eicosatetraenoic acid which potentially is a contributing factor for thrombosis in ischemic hearts [34]. Epicardin, TCF21, is a transcription factor crucial for cardiac fibroblasts. In mouse null-model lacking TCF21, hearts fail to produce cardiac fibroblasts, which are essential for proper heart function [35]. GRB7 plays a significant role in the production of stress granules [36] involved in promoting cell survival [37] under various stress conditions.

We observed a distinct down-regulation of HOX genes of which a majority belonging to the HOXC family. HOXC cluster genes have been observed to be up-regulated in subcutaneous compared with omental adipose tissue [38] which is in line with our findings of down-regulation in EAT. Down-regulation of HOX genes including HOXA6, HOXA7 and HOXC6 has been observed in epicardial adipose tissue of patients with atrial fibrillation [39]. Abnormalities in HOX gene expression could potentially lead to structural defects or malformations in the heart, predisposing individuals to atrial fibrillation. The distinct down-regulation of HOX genes in EAT might have implications for the structural and functional differences between EAT and SAT. Among the down-regulated genes are NNAT and NPY1R which are both indirectly associated with obesity. NNAT encodes for a protein localised in the endoplasmic reticulum (ER) and increased expression of this gene has been observed in the blood vessels of obese and diabetic mice, suggesting that it may influence the inflammatory pathway in diabetic vascular disease [40]. NPY1R encodes a neuropeptide receptor involved in appetite regulation [41]. Due to the downregulation in EAT it is likely that these functions are not present in EAT.

Notably, expression of the common pro-inflammatory genes TNF-α, CCL2, IL1B was not significantly different between EAT and SAT, which is in line with an earlier study from our group demonstrating that systemic inflammation in humans activates the nuclear factor-κB regulatory pathway with a similar pattern of gene expression of pro-inflammatory cytokines in omental and subcutaneous adipose tissue [11]. However, up-regulation of IL18, CXCL1, and CCL5 suggests an ongoing chronic inflammation in the EAT. An up-regulation of these genes could result in recruitment of immune cells to the EAT with subsequent effects at the underlying myocardium. Indeed, in patients with HFpEF increased EAT is associated with proteomic markers of inflammation and endothelial dysfunction and cardiac functional and structural impairments [42]. Also the EAT secretome through downregulated gesolin (GSN) may activate macrophages and inflammation and play a role in development of atrial fibrillation post CABG [43]. Our findings revealed a notable upregulation of pro-inflammatory genes and downregulation of key adipokine genes, including LEP (Leptin), ADIPOQ, IL6, SERPINE1, and RBP4. This imbalance between pro-inflammatory and anti-inflammatory mediators can be explained by the compromised inflammatory state of the tissue which may directly influence the myocardial environment.

Meta-analysis of EAT versus SAT in patients with coronary artery disease based on qPCR data has revealed up-regulated genes in EAT include UCP-1, IL-1β, AGT, ADM, MCP-1, TNF-α, and ADORA1 [44]. Conversely, genes that were down-regulated include ADIPOQ, CATA, and RBP-4 [44]. We observe a lack of overlap in the genes IL-1β, ADM, TNF, and CATA between our study and the referenced meta-analysis; these genes were not identified as significantly up- or down-regulated in our research. A potential explanation for this discrepancy could be the specific patient populations. Our study exclusively focused on patients undergoing CABG surgery, whereas the meta-analysis encompassed both CABG and valve replacement surgery. The inflammation and homeostasis are likely to be different between the two datasets.

### Adipokines in EAT

The adipokines ITLN1, UCP-1 and LRRN4 were found upregulated in EAT. Low levels of ITLN1 in blood plasma have been observed correlating with CAD [45, 46] and obesity [47]. Given its potential anti-inflammatory [48] and insulin-sensitising properties, the up-regulation of ITLN1 in EAT might be related to different phenotypes of heart dysfunction by modulating inflammation, metabolic dysregulation, or endothelial function, all of which are known to contribute to the progression of HF [47, 49]. Thermogenin, UCP-1, is a mitochondrial protein, typically regarded as a marker for brown adipose tissue [50], responsible for thermogenesis. It has been proposed that EAT functions to defend myocardium and coronary vessels against hypothermia through high levels of UCP-1 [51]. UCP-1 has been reported downregulated in EAT compared to SAT in patients with advanced CAD [52], which seemingly contradicts the results in this study. The difference could be explained by various factors, such as, different stages of the disease or heterogeneous patient population, with both studies using a limited sample size. LRRN4 expression has been observed to be similar in donor hearts and hearts with ischemic heart disease, but is reduced in dilated cardiomyopathy [53]. Further research is needed to understand the role of LRRN4 in the development of ischemic heart disease and dilated cardiomyopathy.

### Weighted gene correlation networks

The weighted gene correlation network analysis revealed an overall strong preservation of EAT modules in SAT (Fig. 2b). While these functional networks in EAT share properties with those in SAT, we see a large difference in how these modules are expressed. The distinct expression patterns observed in EAT and SAT modules could reflect the differences in their functional roles, cellular composition, and microenvironment.

The largest module 1 (turquoise) shows a strong overlap with a study deploying the same network analytic methods on left atrial mouse tissue with induced atrial fibrillation [54]. The overlapping module had 28 of the 30 most intramodular connected genes overlapping, including; BNC1, ALOX15, KLK11 and CGN (Fig. 3a). CGN is a component in the intracellular barrier between epithelial cells modulating the barrier function [55]. Although there is no direct connection between CGN and hypertension, its paralogue CGNL1 has been identified as a potential contributor to hypertension and kidney disease [56]. The module showed significant correlation with atrial fibrillation in left atrial tissue and was suggested to be an important regulator of the disease. This finding indicates similar expression patterns between left atrial tissue and epicardial fat tissue. It is in this module that we also found the pro-inflammatory genes CXCL1 and IL18.

Module 5 (green; Fig. 3b) features genes coding for the scaffold protein CARD11 and RHOH as the most connected in the module – both of which have implications in cardiovascular physiology. CARD11 has been shown to modulate apoptosis in cardiomyocytes following myocardial infarction [57]. Up-regulation of CARD11 is associated with an increase in cell death through apoptosis. Gene knockout studies in mice have demonstrated a significant improvement in heart survivability in response to stress when CARD11 is absent [57]. This may have implications for treatment of HF, as inhibiting CARD11 could be a potential target for protecting the heart from further damage. The module also contains the gene RHOH, which has been proposed as a target for reducing the progression of CAD due to its ability to modulate vascular inflammation. Down-regulation of RHOH has been shown to increase the expression of IL6 [58], which is linked to both acute and chronic inflammation [59]. The KEGG pathway enrichment analysis revealed T Cell Receptor Signalling and Th1 and Th2 cell differentiation which are involved in pathways critical in mediating inflammatory responses and are central to the immune system's response to inflammation, which is a fundamental process in many cardiovascular diseases.

GO analysis of module 21 (dark red; Fig. 3d) reveals genes involved in the amylin receptor signalling pathway. Amylin is a peptide hormone that is typically co-secreted with insulin in the pancreas and has been demonstrated to induce vasodilation, effectively reducing blood pressure, as observed in rabbits when administered intravenously [19]. The module also shows an enrichment of genes involved in the assembly of adherens junctions and cell junctions, suggesting the module playing a role in preserving structural integrity and functionality in EAT.

In module 7 (black; Fig. 3e) we observed an enrichment of genes involved in cellular response to zinc ion and regulation of p38MAPK cascade. The latter is a key signalling pathway that mediates in cellular response to stress and inflammation. Notably, one of the hub genes within this module is the downregulated BMP2 which is also found the regulation of p38MAPK cascade. This could potentially affect the cellular responses to stress and inflammation mediated by the p38MAPK cascade.

The third largest module 4 (yellow) contains a large number of adipose tissue specific genes of which several involved in fatty acid oxidation (Fig. 3f). The module contains the cardio-protective cytokine adiponectin, ADIPOQ, contributing to fatty acid combustion [60]. Expression of ADIPOQ has proven downregulated in EAT in patients with CAD [61] where it is believed to favour accumulation of free fatty acids in myocardium overcombustion [60]. Further, fatty acid β-oxidation is observed to be downregulated in mice right ventricular overload in favour of glycolysis [62]. A leading hub gene of the module is PEX19 which is an important factor in peroxisomal membrane genesis, a critical aspect in maintaining efficient fatty acid β-oxidation as peroxisomes are involved in the initial steps of breaking down long-chain fatty acids. While the full range of its functions is not yet known, recent studies have revealed that PEX19 extend its functional role beyond peroxisomal biogenesis, aiding in the sorting of certain membrane proteins to other cellular organelles, including the endoplasmic reticulum (ER), lipid droplets, and mitochondria. [63]. Its functioning role in this module suggests a possible interaction with the adipose specific genes within the module. 

Module 2 (blue; Fig. 4a) contains the phospholipid and free cholesterol transfer protein PLTP, which helps in regulating the size and composition of high density lipoproteins (HDL), known for their protective effects against CAD [64]. Elevated protein levels of PLTP have been reported in patients with CAD [65, 66]. The gene CAPNS1 found among the hub genes with significantly different expression is essential for calcium activated protease calpain activity and function [67]. Calpain proteins are Ca^2+^-dependent intracellular cysteine proteases believed to be activated by Ca^2+^ overload in diseased hearts [68]. In mice with pressure overload-induced heart and CAPSN1 deficiency, alterations in the plasma membrane have been observed, indicating Calpain having a protective effect on the heart [68].

Module 9 (magenta; Fig. 4b) contains several ribosomal protein coding genes and was highly preserved in the analysis suggesting that these genes are crucial for maintaining cellular functions across the tissues. The module is enriched in SRP-dependent co-translational protein targeting to membrane (GO:0006614), genes, indicating its importance for the proper functioning of the cell's endoplasmic reticulum (ER) and likely its role in maintaining the cell's overall health and viability which also reflects its high preservation in SAT (Fig. 2b).

The least preserved module12 (tan) contains FLT4, SEMA3A, CCL21, TBX1 and PROX1 which are all involved in processes related to angiogenesis, cell signalling and immune response. The distinction as the least preserved module suggests that these genes may undergo different expression patterns in EAT relative to SAT. This variance could have implications for understanding the unique role and maybe pathological conditions of EAT. FLT4, also known as VEGFR-3, has been observed to regulate inflammation and metabolic homeostasis in white adipose tissue of obese mice. Blocking FLT4 signalling alleviates inflammation in white adipose tissue in mouse models of obesity, indicating a potential role for FLT4 in the immune response of adipose tissue. SEMA3A has been found to influence cardiac inflammation following myocardial infarction by promoting the resolution of inflammation and improving cardiac function and healing after myocardial infarction in mice [69]. Although, the expression of SEMA3A in this study is not significantly different, its role in modulating the inflammatory response and promoting tissue repair remains noteworthy as it is part of this module. The module also includes ITLN1, ADAM23 and PTX3. ADAM23 is typically found in elevated expression levels in heart tissue [18] and has been suggested to be suppressing cardiac hypertrophy through focal adhesion kinase-protein kinase B pathway inhibition [70], which is involved in regulating cell growth, survival and proliferation. The inhibition of this pathway may prevent excessive growth in cardiac muscle cells. Moreover, PTX3 is produced in several tissues in response to inflammatory stimuli but is also involved in angiogenesis and tissue remodelling, both of which are essential processes in wound healing and tissue repair. High plasma levels of PTX3 have been found in patients with chronic HF and has been proposed as a biomarker for prognosis and inflammatory status of chronic HF [71].

Module 31 (paleturqiouse; Fig. 3c) was found to be associated with cytokine production in inflammatory response. Chronic inflammation in adipose tissue is a risk factor in developing insulin resistance and type 2 diabetes [72]. In addition to its role in metabolic disorders, chronic inflammation is also implicated in the pathogenesis of cardiovascular diseases, such as CAD [73] and HF [3]. One of the factors involved in cytokine production is TLR4, which was identified in the module. TLR4 is crucial for activating the immune system's response to various pathogens and endogenous signals of tissue damage [74]. It plays a role in atherosclerosis via its detrimental effect on endothelial cells and fibrosis of vascular smooth muscle cells. By dampening the TLR4-mediated inflammatory response, it may be possible to slow down or prevent the progression of atherosclerosis and reducing the risk of CAD [75].

### Module to trait correlations

Significant correlation of module Eigen gene (module principal component 1) to echocardiographic parameters was found in seven modules, namely modules 33 (dark olive green), 21 (dark red), 3 (brown), 12 (tan), 19 (light yellow), 2 (blue) and 26 (dark orange), displayed in Fig. 2c. The modules’ correlation to physical traits was largely on relative wall thickness, deceleration time, end diastolic LV wall thickness and BMI, all factors related to different phenotypes of HF.

Module 2 (blue) exhibits a strong positive correlation with relative wall thickness (0.68, *p*-value: 0.0001) which may influence the development and remodelling of the heart wall. Among the hub genes of the module we find CAP1, involved in the regulation of the actin cytoskeleton and important for maintaining the cell shape and function, ACTR1A involved in the regulation of actin polymerisation and TUBA1C, which forms the building blocks of microtubules, an essential component of the cell’s cytoskeleton. The module also demonstrates a negative correlation to deceleration time (–0.6, *p*-value: 0.006). A shorter deceleration time is often indicative of increased LV filling pressure and impaired LV relaxation seen in HFpEF. Together, these correlations may reflect mechanisms leading to structural cardiac changes and impaired diastolic function, potentially contributing cardiac dysfunction. The genes within this module could be acting through pathways that both promote hypertrophic remodelling and impair the relaxation capability of the heart. In contrast, module 3 (brown), 21 (dark red), and 23 (dark turquoise) show negative correlations with relative wall thickness. These modules may represent different aspects of pathways, potentially counteracting the effects observed in module 2 or highlighting alternative pathways that influence heart structure and function. The negative correlation suggests these modules might be involved in pathways that either prevent hypertrophic remodelling or enhance the heart's relaxation capabilities.

Module 33 (dark olive green) displayed a strong negative correlation with BMI (–0.71, *p*-value:0.0007). The hub gene for the module, RAB11B, is involved in insulin secretion and the cellular response to glucose stimulus. NDUFB7, another hub gene found in the module, is part of the mitochondrial electron transport chain, crucial for cellular energy metabolism potentially influencing BMI regulatory pathways. The correlation between module 33 and BMI may suggest a link between the genes in this module and metabolic processes or insulin sensitivity. Module 33 is the most preserved module between EAT and SAT and without significantly enriched differentially expressed genes, indicating that it is a highly conserved gene network module that plays a fundamental role in both fat depots. The absence of enriched differentially expressed genes in module 33 suggests that its genes are vital for adipose tissue's basic structure and function, regardless of the fat type. These genes may participate in crucial processes like lipid metabolism, adipogenesis, or adipocyte differentiation in both epicardial and subcutaneous fat. The correlation between module 33 and BMI suggests that the module could be involved in functions related to body weight regulation, adiposity, or energy homeostasis.

### Clinical implications

The roles of subcutaneous and epicardial fat are clearly different. We highlight the gene expression difference in search of potential modifiers of HF progress. In pre-HF patients (ACC/AHA stage B) with signs but no symptoms of HF compared to patients with no signs of HF (ACC/AHA stage A) we clearly found 18 different genes associated with stage B. The true implications of our findings should be corroborated in other studies since HF ACC/AHA stage B patients are common and carry a considerable risk for progression to symptomatic HF.

## Conclusion

In patients undergoing coronary artery bypass surgery, we identified seven gene modules in epicardial adipose tissue (EAT) that correlated with echocardiographic characteristics of left ventricular (LV) dysfunction. These modules were associated with the relative wall thickness, deceleration time, e', and BMI. One module was notably less preserved in epicardial adipose tissue compared to subcutaneous adipose tissue, suggesting a unique role in the heart’s epicardial fat and marking it as a target for further study. Additionally, one module with the driving genes PLTP and CAPNS1 showed a significant connection with end diastolic LV posterior wall thickness, a key predictor of sudden cardiac arrest in hypertrophic cardiomyopathy, potentially offering insights into its molecular mechanisms and associated risks. Further, our analysis revealed DEGs in EAT between patients with no signs of HF (ACC/AHA stage A) and pre-HF patients (ACC/AHA stage B), indicating distinct molecular signatures that could contribute to the pathophysiology of early HF.

## Methods

### Patients

Participants in the study were individuals scheduled for elective Coronary Artery Bypass Grafting (CABG) who did not require simultaneous valve surgery. These participants experienced angina pectoris, with some having a history of myocardial infarction. During the CABG procedure, cardiac biopsies were collected before the initiation of the heart–lung machine. Prior to the surgery, all participants underwent a baseline evaluation 4–8 weeks earlier, which included clinical assessments, echocardiography, and blood tests that measured natriuretic peptides.

*Descriptive data* are presented as median and quartiles (Q1;Q3) or number (%), and comparisons between groups were performed by Kruskal–Wallis and chi-square tests as appropriate.

### Tissue collection

Epicardial adipose tissue biopsies were taken from patients undergoing CABG before cardiac arrest and stored at –70 °C for mRNA analysis. Patients were prepared for surgery with standard clinical routines, including placement of central, arterial, and peripheral lines. A midline sternotomy was performed and one or two mammary arteries were procured for use as conduits [64]. The subcutaneous adipose tissue biopsies were taken deeply from the side of the median sternotomy incision.

### RNA extraction and sequencing

Total RNA was extracted with the RNeasy Lipid Tissue Mini Kit (#74,804, Qiagen) and used to prepare RNA libraries for sequencing with poly-A selection and the Illumina RNA strand-specific TruSeq Stranded mRNA Sample prep kit with 96 dual indexes (Illumina, CA, USA). The protocols were automated on an Agilent NGS workstation (Agilent, CA, USA) with purification steps as described [65, 66].

Samples were clustered 'cBot' and sequenced on NovaSeq6000 (NovaSeq Control Software 1.6.0/RTA v3.4.4) with a 2 × 151 setup using 'NovaSeqXp' workflow in 'S4' mode flowcell. The Bcl to FastQ conversion was performed using bcl2fastq_v2.19.1.403 from the CASAVA software suite. The quality scale used was Sanger / phred33 / Illumina 1.8 + .

### RNA-seq analysis

Full transcriptome sequencing was performed for each biopsy. The raw paired-end reads were first checked for quality using FastQC (version 0.11.9) to identify any potential outliers or quality issues.. After this, the reads were mapped against the human genome (GRCh38) using STAR aligner (2.7.8) (Supplementary Figure S1). Reads that mapped to the exons of the coding genes were counted using HTSeq (0.12.4) [76], in union mode. Genes with lower combined counts of 100 was omitted. DESeq2 [77] was used to analyse the differential expression using SF as reference in a paired model. Genes with an adjusted *p*-value (false discovery rate) ≤ 0.05 was marked as significant. Normalisation of the count data was done using DESeq2’s median of ratios.

### Human Protein Atlas

Genes with least four-fold higher mRNA levels in adipose specific tissues were extracted from the Human Protein Atlas [18], totalling 196.

#### Weighted gene correlation networks

Based on the principle that genes with similar expression patterns share the same pathway or functional mechanisms we utilised Weighted Gene Correlation Analysis (WGCNA). The analysis was performed on variance stabilised and filtered EAT data containing median gene counts ≥ 50. Genes with a high correlation (> 0.5) to beta-globin component of hemoglobin (from blood contamination in EAT biopsies) were excluded. A scale free topology (R ≥ 0.8) was reached with a soft power threshold of 18. Modules were merged with WGCNA’s function mergeCloseModules using a cut height of 0.2, meaning modules of eigengene correlation above 0.8 were merged. Adjacency was selected as “signed”, meaning two negatively correlated genes are considered unconnected in the network. A gene–gene correlation matrix was calculated with a biweight midcorrelation (bicor) upon which a topological overlap measure (TOM) matrix, representing the connectivity of the network, was calculated. Gene modules of at least a size of 25 was extracted based on a hierarchical clustering using the flashClust function and modules sharing a correlation of 80% or higher were merged with the function mergeCloseModules,

#### Module preservation and correlations

A pair-wise gene comparison between test-set (EAT) and reference set (SAT) was calculated with the WGCNA function ‘modulePreservation’ [78] representing the preservation in Z_Summary_ score. The function calculates the network density and connectivity preservation of a reference set to a test set in the average value Z_Summary_ score. Preservation is regarded as strong for Z_Summary_ > 10 and weak for Z_Summary_ < 2 as by the creators of WGCNA [78]. Significant overlap of DEGs (–0.5 ≤ log_2_fold ≤  + 0.5) in modules and module to trait correlation were examined with a Fisher exact test with the inbuilt WGCNA function ‘overlapTable’. Overlap was regarded as significant for *p*-values ≤ 0.05.

#### String network comparison

Comparing the modules to the STRING database of protein–protein interactions involved examining whether the list of genes representing the modules had documented interactions in the STRING database. The score presented for each interaction utilised a combined score approach. This score is calculated by combining probabilities the various types of interaction in the database, such as co-expression, co-localisation and text mining. The suggested thresholds for combined scores, as recommended by the creators of STRING, include low (0.15), medium (0.40), high (0.70), and highest (0.90) confidence levels to indicate the strength of the protein–protein interactions. The lookup for protein–protein interactions was performed using STRING's API.

#### Functional analysis

Functional enrichment analysis of GO biological process terms was performed using Enrichr [79] on all modules. Terms were selected on p-values corrected with the Benjamini–Hochberg method for multiple hypotheses testing. Adjusted *p*-values were considered as significant for values ≤ 0.05.

#### Ethics statement

The study was carried out in accordance with the Declaration of Helsinki and was reviewed and approved by the regional Ethics Committee in Stockholm. All patients provided written and oral informed consent prior to their inclusion in the study.

#### Limitations

The limited amount of biopsy material available for our study required us to use the entire biopsy for deep RNA sequencing, which improved the sensitivity of the analysis but precluded us from performing any validation experiments. Additionally, our sampling cohort predominantly consists of male patients (95%) which introduces a significant gender bias. Given this composition, we acknowledge that the gene expression differences reported between EAT and SAT primarily reflect the male perspective. A larger cohort of patients, will add more insight into differences in gene expression between these two groups as well as difference between HF conditions of HFpEF and HFrEF.

A larger sample size of patients would also make it possible to control for confounding variables such as age, gender and comorbidities which could possibly provide better results. This has been a bioinformatics study which has not been experimentally validated. Further research is needed to verify the results published in this this report.

## Supplementary Information

Supplementary Material 1.

Supplementary Material 2.

Supplementary Material 3.

Supplementary Material 4.

Supplementary Material 5.

Supplementary Material 6.

Supplementary Material 7.

## Data Availability

RNA-seq data have been deposited in the EMBL-EBI ArrayExpress database (www.ebi.ac.uk/arrayexpress) under accession number E-MTAB-14321.
