# Supplementary material for: Characteristics of gene expression in epicardial adipose tissue and subcutaneous adipose tissue in patients at risk for heart failure undergoing coronary artery bypass grafting
Source: BMC Genomics. 2024 Oct 7;25:938. doi: 10.1186/s12864-024-10851-9 (PMC11457432; doi:10.1186/s12864-024-10851-9)
Supplement: Supplementary file 1 — Supplementary Material 1. [file 12864_2024_10851_MOESM1_ESM.pdf]

Supplementary Material to:

Characteristics of gene expression in epicardial adipose tissue and subcutaneous adipose tissue in patients at risk for heart failure undergoing coronary artery bypass grafting

Christoffer Frisk[1], Mattias Ekström[2,3], Maria J Eriksson[4,5], Matthias Corbascio[4,6], Camilla Hage[7,8], Hans Persson[2,3], Cecilia Linde[7,8] and Bengt Persson[1]

[1]Department of Cell and Molecular Biology, Science for Life Laboratory, Uppsala University, S-751 24 Uppsala, Sweden; [2]Karolinska Institutet, Department of Clinical Sciences, Danderyd Hospital, S-182 88 Stockholm, Sweden; [3]Danderyd Hospital, Department of Cardiology, S-182 88 Stockholm, Sweden; [4]Karolinska University Hospital, Department of Clinical Physiology, S-171 76 Stockholm, Sweden; [5]Karolinska Institutet, Department of Molecular Medicine and Surgery, S-171 77 Stockholm, Sweden; [6]Karolinska University Hospital, Department of Thoracic Surgery, S-171 76 Stockholm, Sweden; [7]Karolinska Institutet, Department of Medicine, S-171 77 Stockholm, Sweden; [8]Karolinska University Hospital, Heart and Vascular Theme, S-171 76 Stockholm, Sweden.

Supplementary Tables

Supplementary Table S1.

Differentially expressed genes (DEGs) across the various phenotypes and tissues. The spreadsheet file consists of two sheets. The first sheet lists DEGs of EAT vs SAT, and the second sheet lists of DEGs in EAT between Stage A and stage B.

Supplementary Table S2.

The spreadsheet file contains sheets for each gene module identified in the study, labelled as “1 (turquoise)” for module 1, “2 (blue)” for module 2, etc. Each sheet lists the genes in that module along with the following columns: BaseMean (average expression level), log2 Fold Change (expression difference between EAT and SAT), lfcSE (standard error of log2 fold change), stat (test statistic), p-value (significance of expression difference), and Adjusted p-value (corrected for multiple testing).

Supplementary Figures

Supplementary Figure S1. **(a)** Mapping Status per Sample: Stacked bar plot representing the distribution of RNA-seq reads across four mapping categories: "Uniquely mapped," "Mapped to multiple loci," "Mapped to too many loci," and "Unmapped: too short." Each bar corresponds to a sample, with the total height of each bar representing the total number of reads (in millions). The colour-coded sections within each bar show the proportion of reads falling into each mapping category. The majority of reads are uniquely mapped, indicating the high specificity of the RNA-seq data. **(b)** Gene Body Coverage: Line plot showing the normalised read coverage across the length of gene bodies for each RNA-seq sample. The x-axis represents the relative position along the gene body, from 5' to 3' end, while the y-axis shows the normalised coverage. A slight 5' bias is present, with coverage peaking around the middle of the gene body, followed by a decrease towards the 3' end. This pattern indicates consistent coverage across the gene bodies, with no significant degradation observed, supporting the overall integrity of the RNA samples.

Supplementary Figure S2. Visualisation of WGCNA modules in STRING. Each subplot represents the protein–protein interaction network for one of the WGCNA modules, with nodes coloured according to their respective module membership. Node labels indicate gene names, and edges represent determined protein–protein interactions. Network layout is determined by the Kamada–Kawai algorithm, which positions nodes to minimise edge crossings. For readability only the top 15 most connected genes (highest degree) are labelled.

Supplementary Figure S3. Comprehensive visualisation of GO enrichment terms and hub gene expression levels. The bar plot displays GO enrichment for all significant terms, with the top x-axis representing the –log(padj) values plotted as a line and the bottom x-axis indicating the percentage of involved genes. The GO terms are ordered by EnrichR's combined score. This supplementary figure provides a more detailed view of the GO enrichment analysis, including all significant terms, compared to the main figure (Figure 3) that displays the subset of top six terms based on combined score.

Supplementary Figure S4. KEGG pathway enrichment analysis results for modules with significantly enriched pathways (adjusted p-value ≤ 0.05). Each subplot represents a different module, colour-coded based on its assigned module colour. The enriched pathways are sorted by their combined score, reflecting their significance and relevance within the corresponding module. The horisontal bars represent the percentage of genes involved in each pathway. A secondary axis (top) shows the corresponding –log(adjusted p-value) for each pathway, with the red dashed vertical line marking the significance threshold of $p = 0.05$ (–log(0.05)).
