# Supplementary figures and images for: Characteristics of gene expression in epicardial adipose tissue and subcutaneous adipose tissue in patients at risk for heart failure undergoing coronary artery bypass grafting
Source: BMC Genomics. 2024 Oct 7;25:938. doi: 10.1186/s12864-024-10851-9 (PMC11457432; doi:10.1186/s12864-024-10851-9)

**a**

Mapping Status per Sample

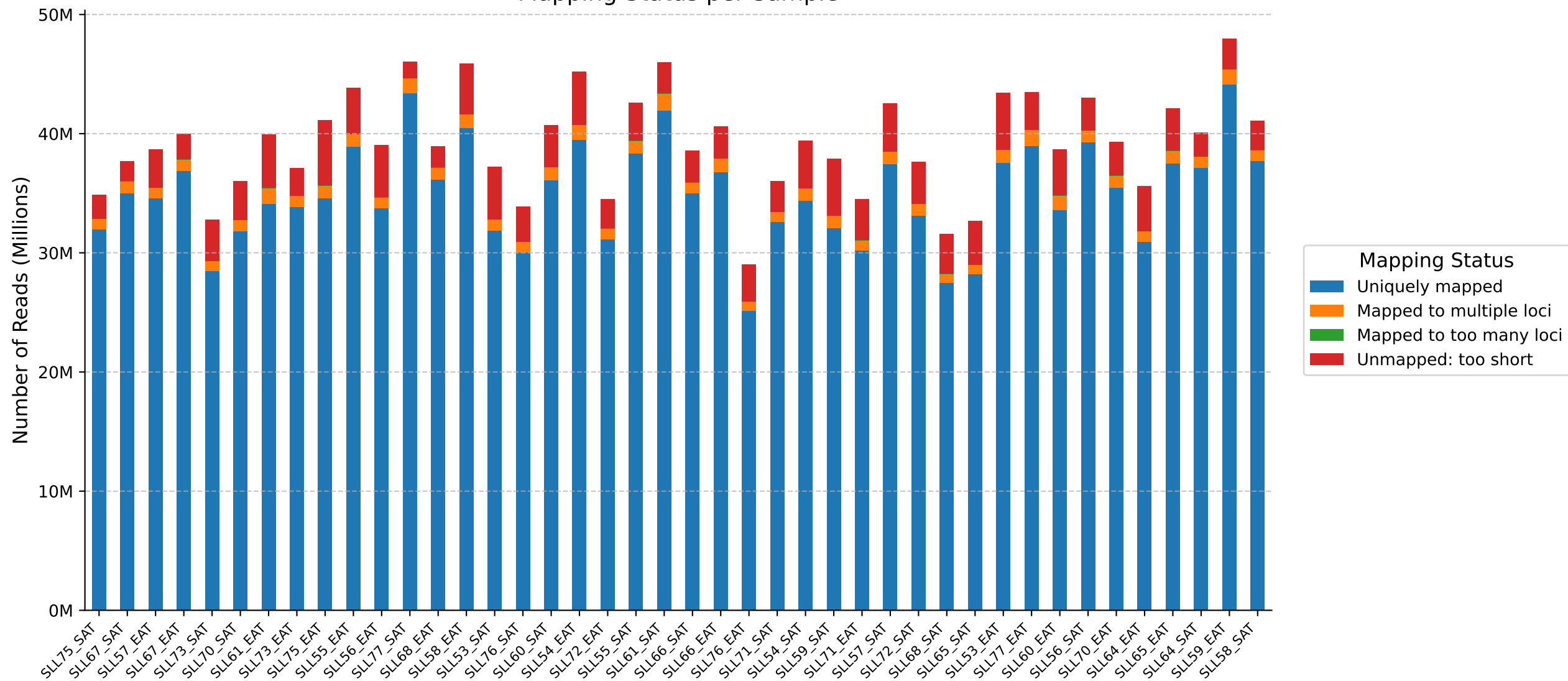**b**

Gene Body Coverage

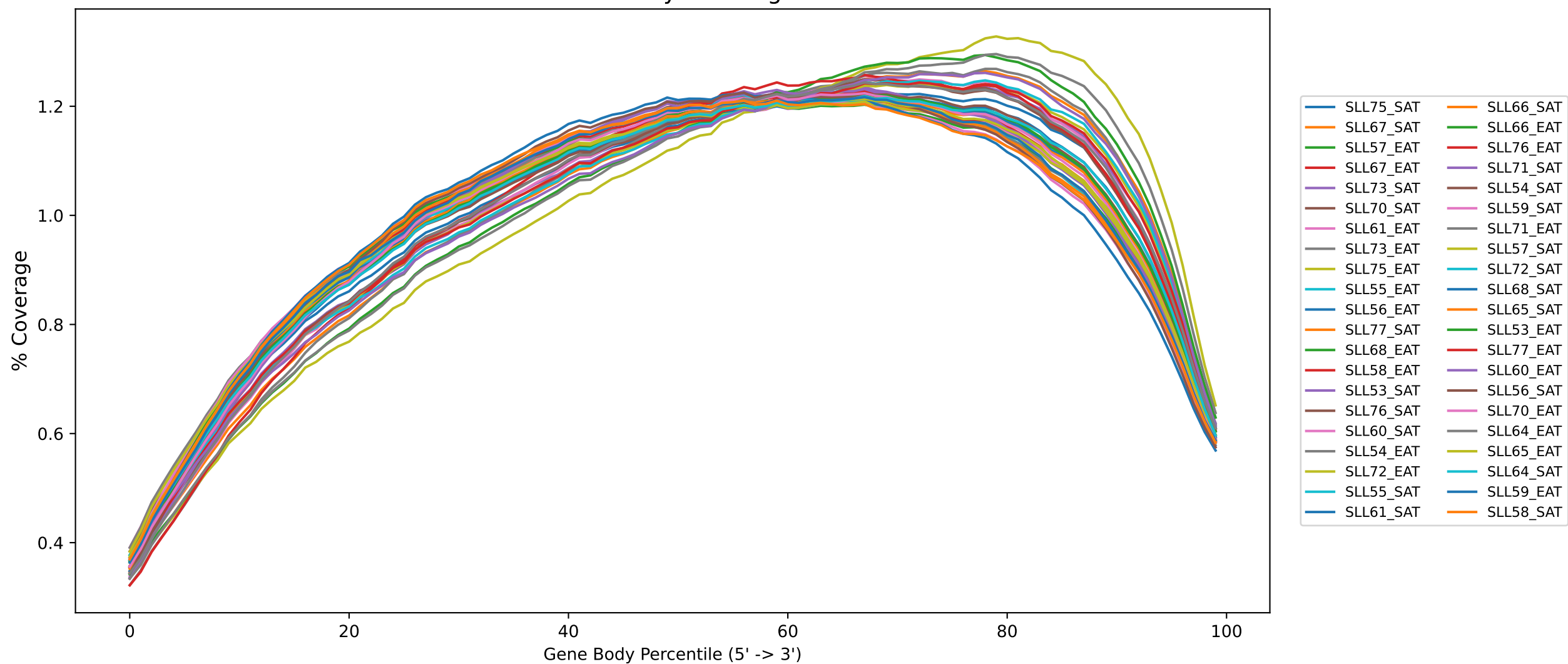

Supplement: Supplementary file 2 — Supplementary Material 2. [file 12864_2024_10851_MOESM2_ESM.pdf]

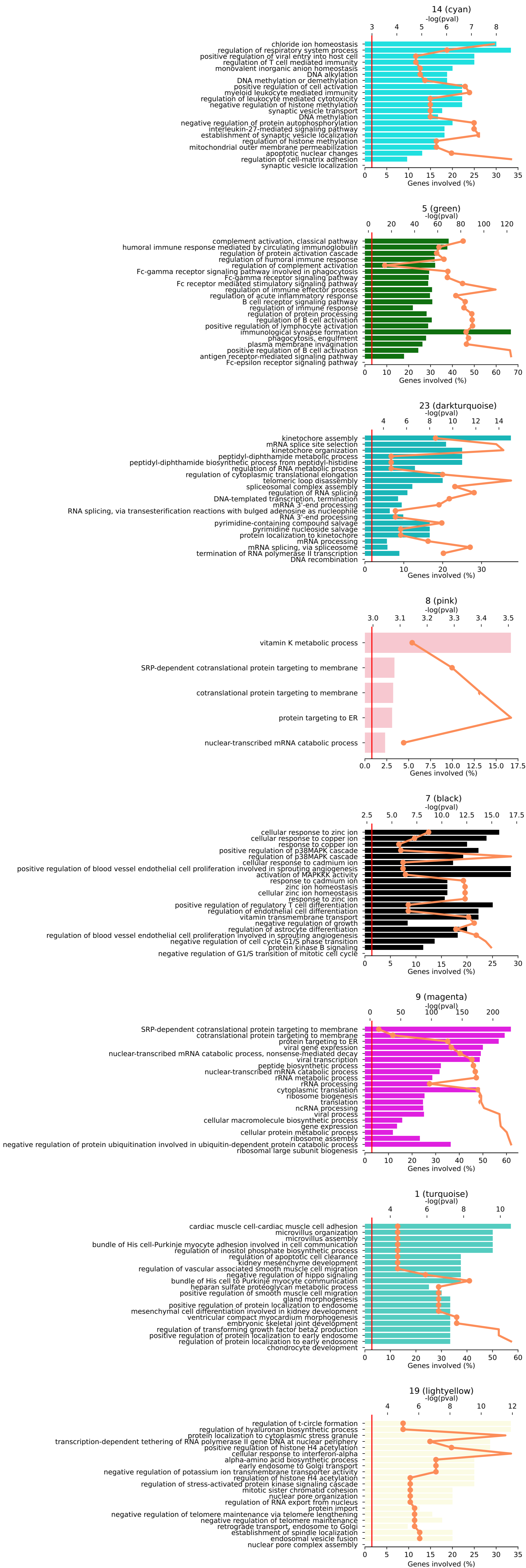

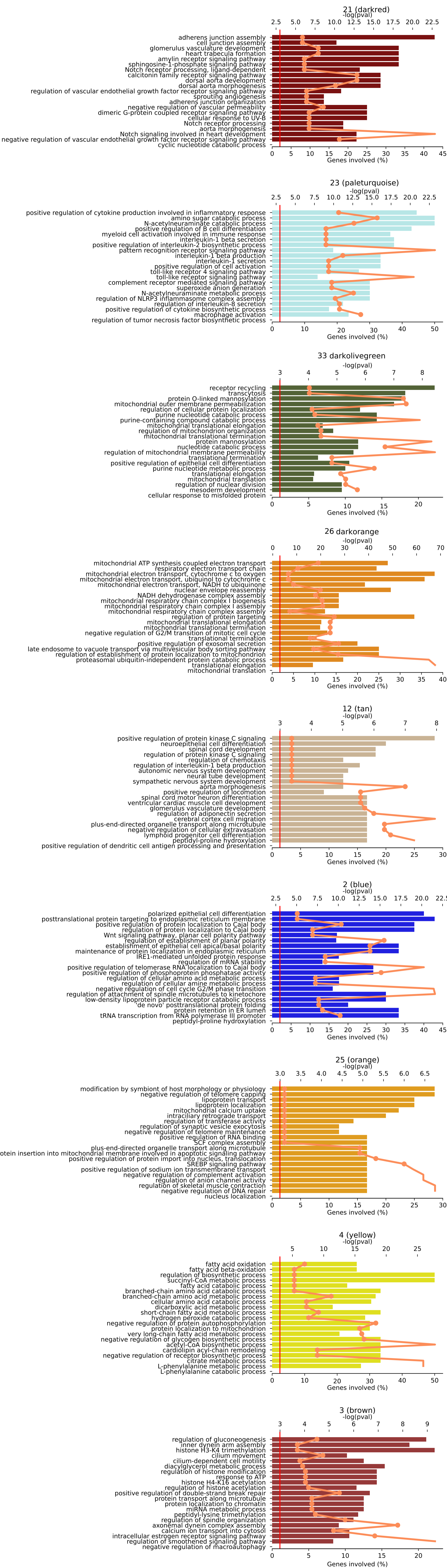

Supplement: Supplementary file 4 — Supplementary Material 4. [file 12864_2024_10851_MOESM4_ESM.pdf]

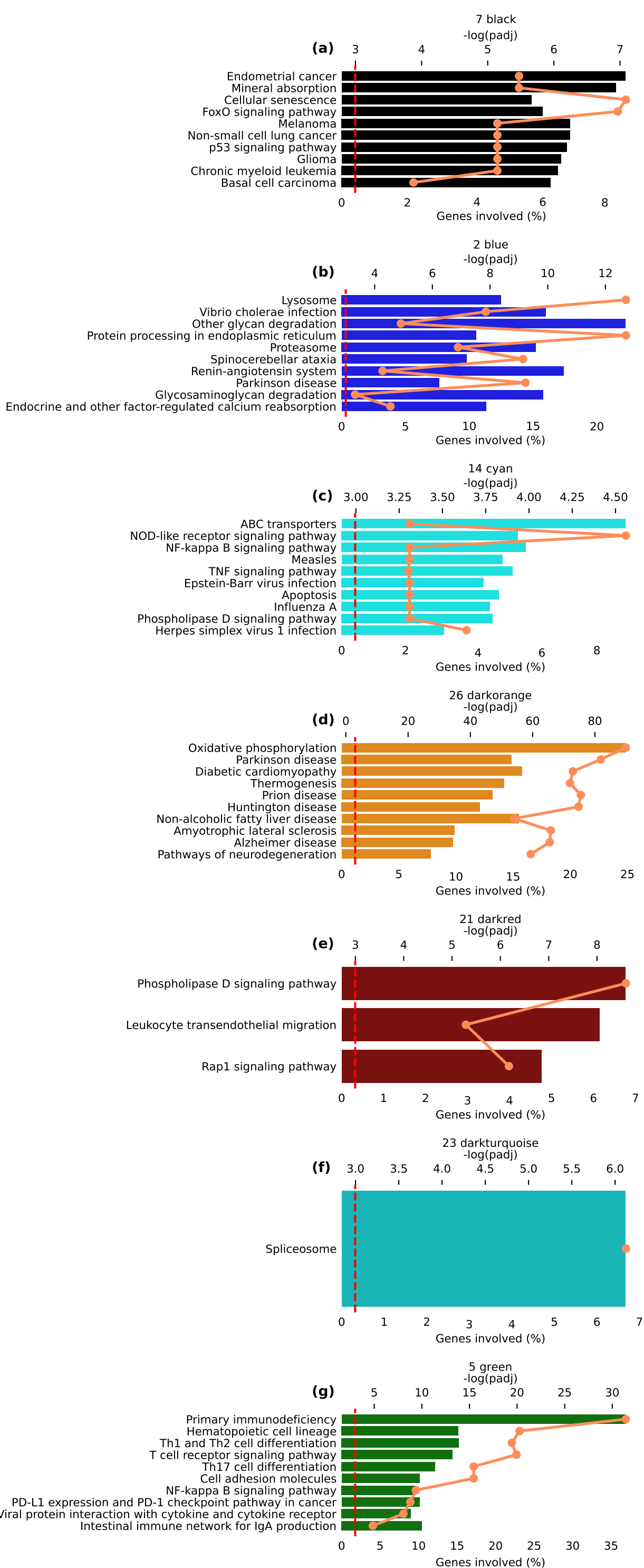

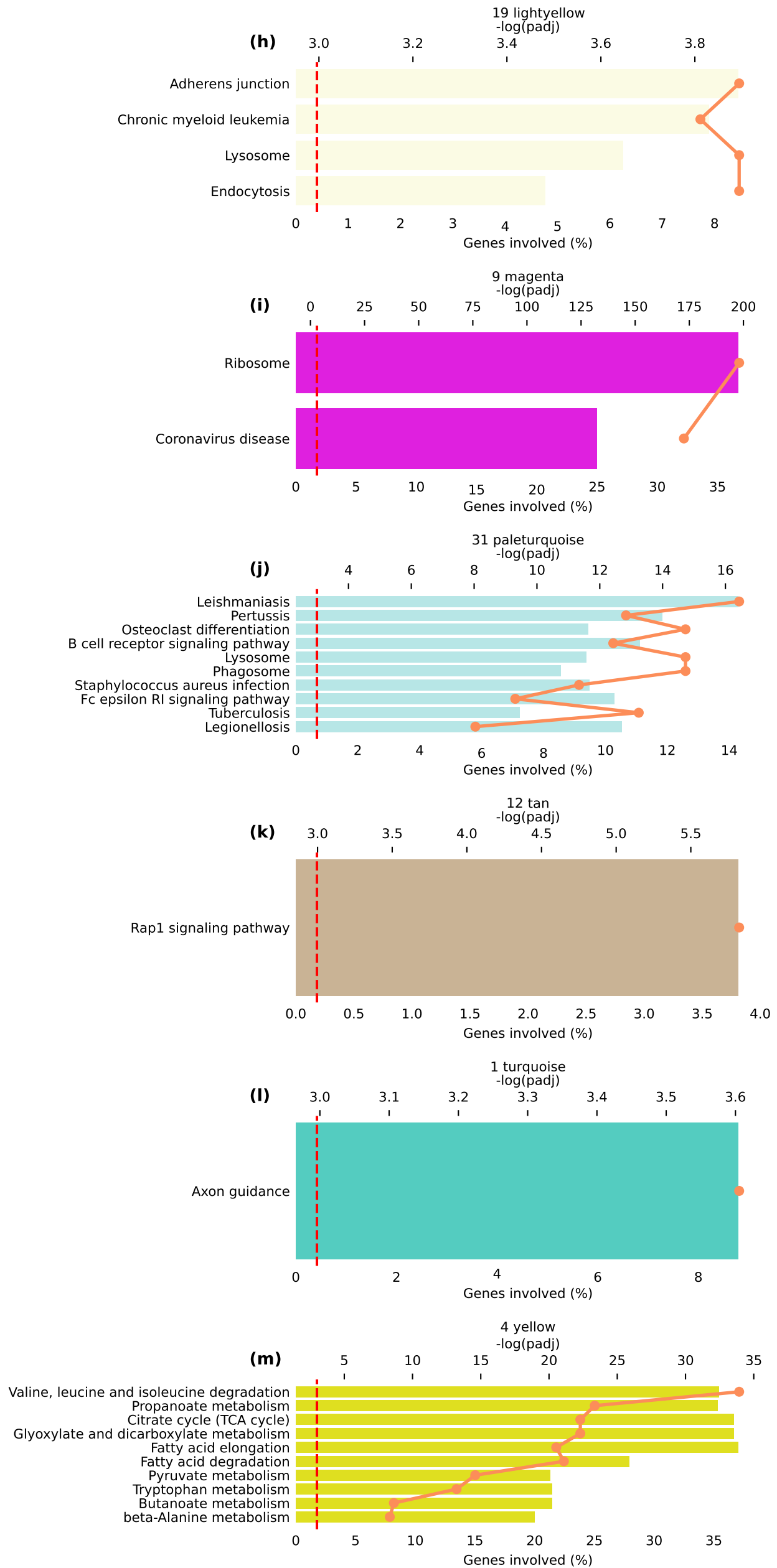

Supplement: Supplementary file 5 — Supplementary Material 5. [file 12864_2024_10851_MOESM5_ESM.pdf]
