# Supplementary material for: Characteristics of gene expression in epicardial adipose tissue and subcutaneous adipose tissue in patients at risk for heart failure undergoing coronary artery bypass grafting
Source: BMC Genomics. 2024 Oct 7;25:938. doi: 10.1186/s12864-024-10851-9 (PMC11457432; doi:10.1186/s12864-024-10851-9)
Supplement: Supplementary file 3 — Supplementary Material 3. [file 12864_2024_10851_MOESM3_ESM.pdf]

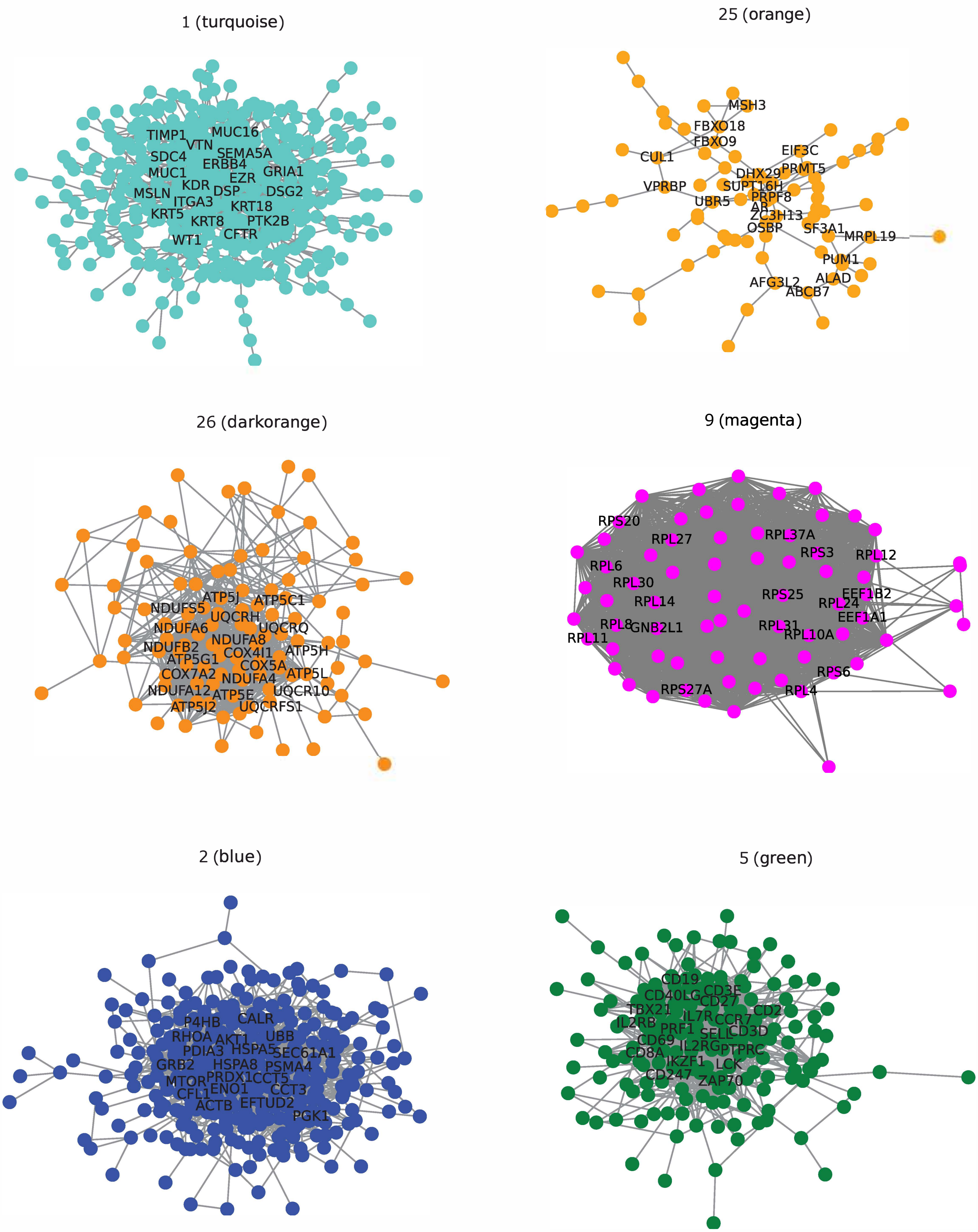
1 (turquoise)
TIMP1 MUC16 VTN SDC4 SEMA5A ERBB4 MUC1 GRIA1 EZR KDR DSP DSG2 MSLN ITGA3 KRT18 KRT5 KRT8 PTK2B WT1 CFTR
25 (orange)
MSH3 FBXO18 FBXO9 CUL1 EIF3C DHX29 PRMT5 VPRBP SUPT16H UBR5 PRPF8 AR ZC3H13 OSBP SF3A1 MRPL19 PUM1 ALAD AFG3L2 ABCB7
26 (darkorange)
ATP5J ATP5C1 NDUFS5 UQCRH NDUFA6 UQCRQ NDUFA8 NDUFB2 ATP5H COX4I1 ATP5G1 COX5A COX7A2 NDUFA4 ATP5L NDUFA12 ATP5E UQCR10 ATP5J2 UQCRFS1
9 (magenta)
RPS20 RPL27 RPL37A RPS3 RPL12 RPL6 RPL30 RPS25 EEF1B2 RPL14 RPL24 EEF1A1 RPL8 GNB2L1 RPL31 RPL11 RPL10A RPS6 RPS27A RPL4
2 (blue)
P4HB CALR RHOA AKT1 UBB PDIA3 HSPA5 SEC61A1 GRB2 HSPA8 PSMA4 PRDX1 CCT5 MTOR ENO1 CFL1 CCT3 ACTB EFTUD2 PGK1
5 (green)
CD19 CD3E CD40LG CD27 TBX21 IL7R CD2 IL2RB CCR7 PRF1 SELL CD3D CD69 IL2RG PTPRC CD8A IKZF1 LCK CD247 ZAP70

4 (yellow)

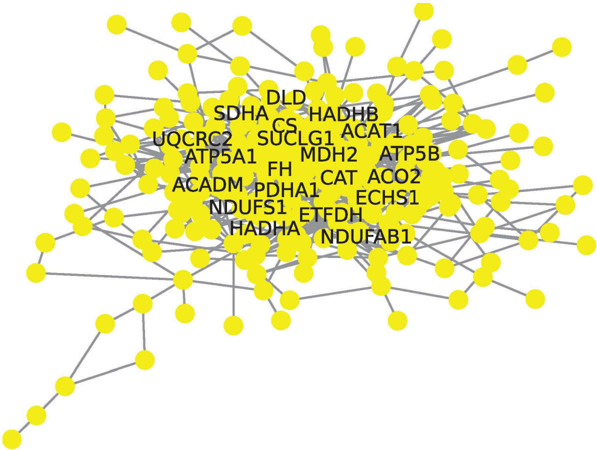

31(paleturquoise)

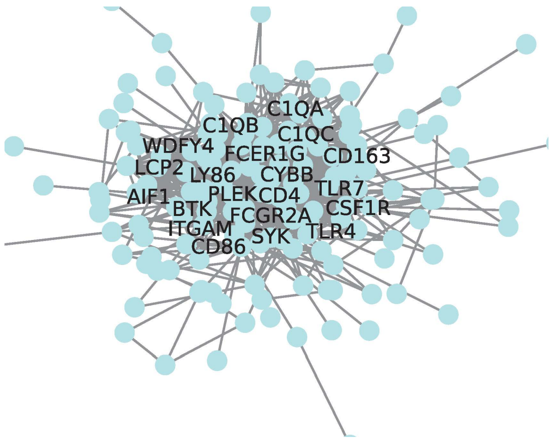

19 (lightyellow)

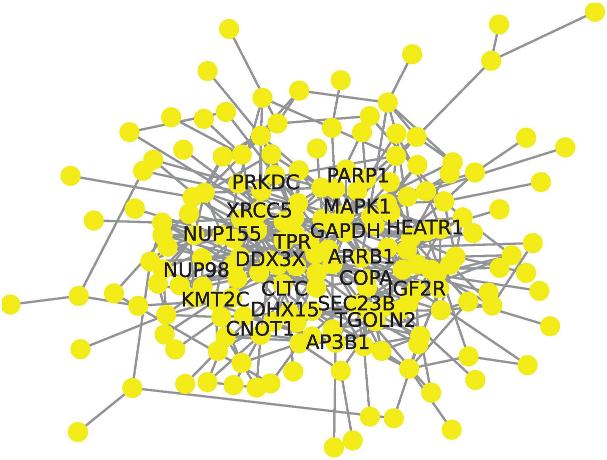

33 (darkolivegreen)

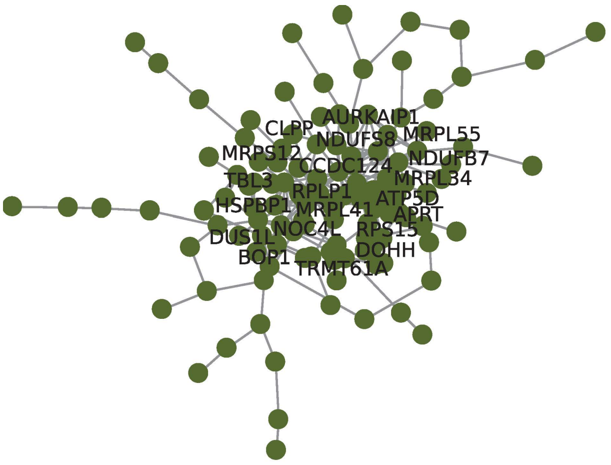

7 (black)

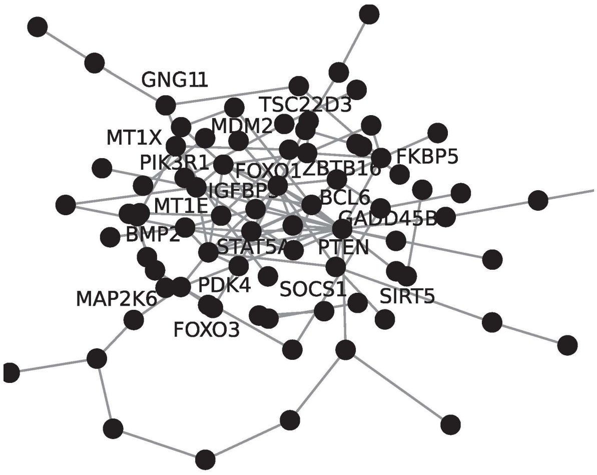

3 (brown)

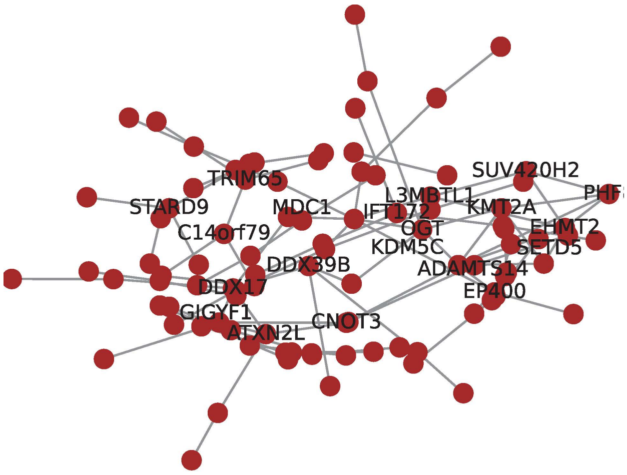

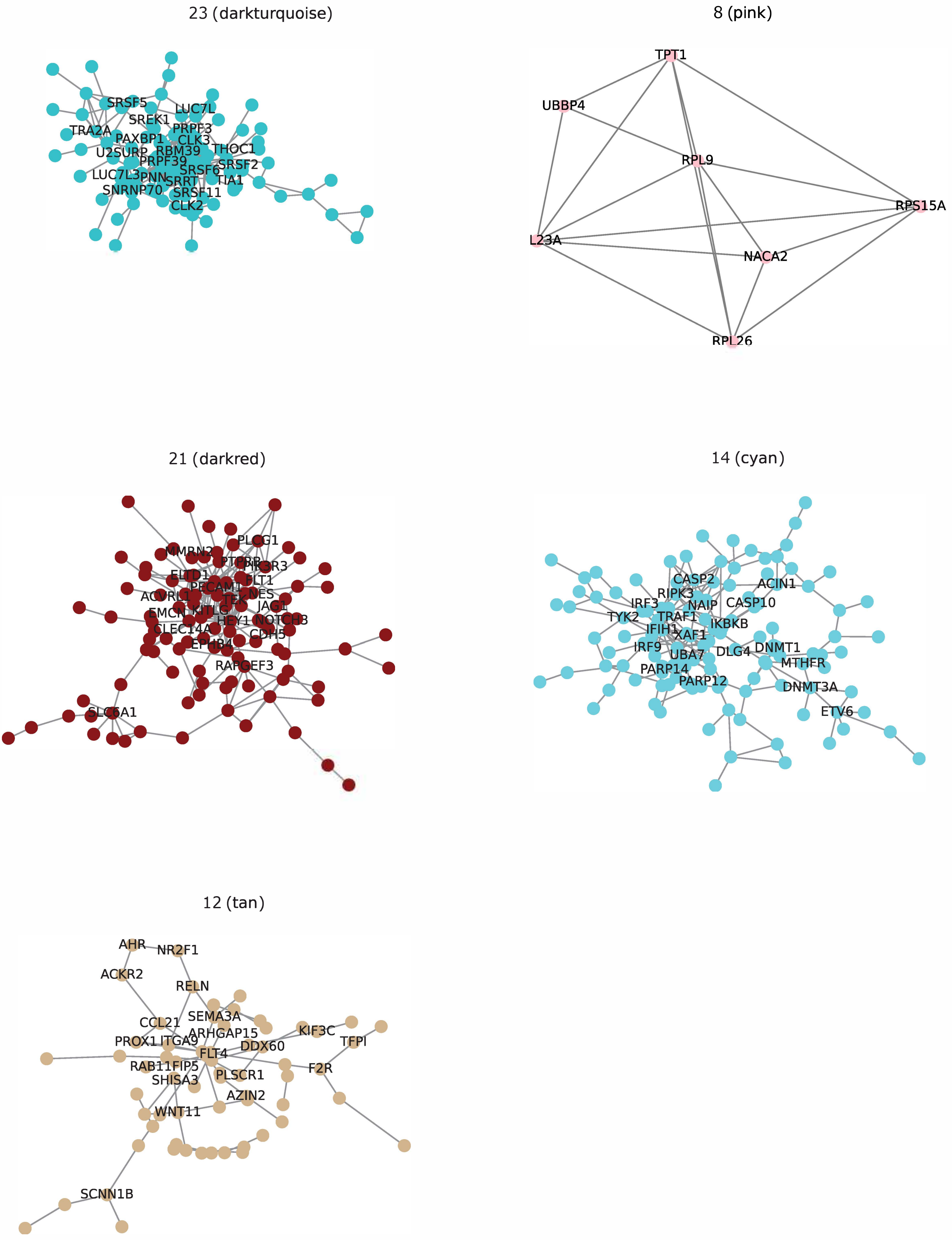

23 (darkturquoise)
SRSF5
LUC7L
SREK1
PRPF3
TRA2A
PAXBP1
CLK3
U2SURP
RBM39
THOC1
PRPF39
SRSF2
SRSF6
LUC7L3
PNN
SRRT
TIA1
SNRNP70
SRSF11
CLK2
8 (pink)
TPT1
UBBP4
RPL9
RPS15A
L23A
NACA2
RPL26
21 (darkred)
PLCG1
MMRN2
PTPRB
PIK3R3
ELTD1
FLT1
PECAM1
ACVRL1
NES
TEK
JAG1
EMCN
KITLG
HEY1
NOTCH3
CLEC14A
CDH5
EPHB4
RAPGEF3
SLC6A1
14 (cyan)
CASP2
ACIN1
RIPK3
IRF3
NAIP
CASP10
TYK2
TRAF1
IKBKB
IFIH1
XAF1
IRF9
DNMT1
UBA7
DLG4
MTHFR
PARP14
PARP12
DNMT3A
ETV6
12 (tan)
AHR
NR2F1
ACKR2
RELN
SEMA3A
CCL21
ARHGAP15
KIF3C
PROX1
ITGA9
DDX60
TFPI
FLT4
RAB11FIP5
F2R
SHISA3
PLSCR1
AZIN2
WNT11
SCNN1B
